# Impact of a multidisciplinary sleep apnea management group clinic on positive airway pressure adherence and patient-reported outcomes: a randomized controlled trial

**DOI:** 10.1007/s11325-025-03319-x

**Published:** 2025-04-04

**Authors:** Sepideh Khazaie, Reena Mehra, Raman Bhambra, Douglas E. Moul, Nancy Foldvary-Schaefer, Robon Vanek, James Bena, Shannon Morrison, Harneet K. Walia

**Affiliations:** 1 Department of Inflammation and Immunity, Lerner Research Institute, Cleveland Clinic, Cleveland, OH USA; 2 Critical Care and Sleep Medicine, Pulmonary and Critical Care Medicine, University of Washington, Seattle, WA USA; 3 Sleep Disorders Center, Department of Neurology, Neurological Institute, Cleveland Clinic, Cleveland, OH USA; 4 Baptist Health, Miami Cardiac and Vascular Institute, Miami, FL USA

**Keywords:** Obstructive sleep apnea, Positive airway pressure, PAP adherence, Patient-reported outcomes

## Abstract

**Introduction:**

Positive airway pressure (PAP) is the mainstay of treatment for obstructive sleep apnea (OSA). However, suboptimal adherence significantly limits its effectiveness. This study examined the impact of a Sleep Apnea Management (SAM) clinic—an innovative, interactive group intervention providing interpersonal support, education, and resources—on PAP adherence and patient-reported outcomes (PROs) compared to usual care.

**Methods:**

Participants with OSA who were newly prescribed PAP therapy and demonstrated suboptimal adherence (defined using CMS criteria during the first two weeks) were randomized to the SAM clinic (*n* = 26) or usual care (*n* = 30) from April 2019 to November 2022 (NCT-03835702). The primary outcome was the change in average daily PAP usage. Secondary outcomes included changes in the Epworth Sleepiness Scale (ESS), Patient Health Questionnaire-9 (PHQ-9), and PROMIS scales from baseline to 1 and 3 months. Baseline-adjusted mixed-effects linear and logistic models estimated differences between and within groups.

**Results:**

Fifty-six participants were enrolled with a mean age of 55 years, 57% female, 63% Caucasian, median AHI of 22.8 (IQR: 9.3,39.6), and median baseline PAP usage of 172 min. After 3 months, the mean (95% CI) SAM clinic daily PAP use was 193 (139, 247) minutes vs usual care at 148 (110, 185) minutes with a mean difference of 46(-8, 99) minutes per day (*p* = 0.093). Within each group, a mean daily difference of 11(-36,57) minutes (*p* = 0.65) in SAM clinic and -32(-75,12) (*p* = 0.15) in the usual care was observed. No significant differences were observed in PROs between SAM and usual care. Within each group, ESS change was -0.7(-2.5,1.2) (*p* = 0.48) in SAM clinic and -2.5(-4.2, -0.83) (*p* = .004) in usual care. Significant decrease was noted in PHQ-9 within both SAM clinic at-2.2(-3.9, -0.4) (*p* = 0.019) and in usual care at -2.3(-4.0, -0.7) (*p* = 0.006). Improvement in PROMIS sleep-related impairment was noted within both groups: SAM clinic at -3.0(-6.2,0.1) (*p* = 0.059) and usual care group at -3.5(-6.4, -0.60) (*p* = 0.019). Similar changes in PAP adherence and PROS were seen at the 1-month follow-up.

**Conclusion:**

The SAM clinic demonstrated trends toward improved PAP adherence and PROs compared to usual care, though differences were not statistically significant, likely reflecting the study’s small sample size and other methodological constraints, larger, adequately powered studies are needed to confirm these findings and further explore the impact of SAM clinics on PAP adherence and patient outcomes.

**Supplementary Information:**

The online version contains supplementary material available at 10.1007/s11325-025-03319-x.

## Introduction

Obstructive Sleep Apnea (OSA) is a highly prevalent sleep disorder characterized by repetitive episodes of upper airway obstruction during sleep, resulting in intermittent hypoxia and sleep fragmentation. It affects an estimated 10–15% of the adult population, with higher prevalence rates in individuals with obesity, hypertension, and other cardiovascular diseases [1–3].OSA is associated with significant health risks, including increased morbidity and mortality due to cardiovascular diseases, metabolic disorders, and impaired cognitive function [4]. The social and economic burdens of OSA are substantial, encompassing direct medical costs, loss of productivity, and reduced quality of life [5].

Positive Airway Pressure (PAP) therapy is the cornerstone treatment for OSA. PAP devices work by delivering a continuous stream of air through a mask to keep the airway open during sleep, thus preventing apneas and hypopneas [6]. Despite its efficacy, adherence to PAP therapy remains a significant challenge. Studies indicate that 40–70% of patients discontinue PAP therapy within the first year of treatment [7, 8]. Poor adherence is often attributed to factors such as discomfort with the mask, pressure intolerance, nasal congestion, and a lack of perceived benefit from the therapy [9].

The importance of PAP adherence cannot be overstated, as it directly correlates with the reduction of OSA-related symptoms and long-term health risks. Adherent patients experience improved daytime alertness, reduced blood pressure, better glycemic control, and overall enhanced quality of life [10]. Therefore, identifying and implementing strategies to improve PAP adherence is critical in the management of OSA.

Group-based clinics have successfully enhanced education and adherence to treatments for various medical conditions, such as diabetes, obesity, and heart failure [11–13]. In the realm of obstructive sleep apnea management, several behavioral and educational approaches have been studied, including telemedicine-delivered education and feedback [14, 15], audiovisual resources [1, 16], structured counseling, and cognitive behavioral strategies [17]. Although these interventions often show improved CPAP acceptance and moderate gains in adherence, most are implemented in a one-on-one or remote format, limiting the immediate troubleshooting and peer support opportunities that a shared medical appointment can provide.

To address these gaps, our institution developed a Sleep Apnea Management (SAM) clinic, which offers a fully multidisciplinary setting where patients receive comprehensive education, troubleshooting, and support from sleep specialists, advanced practice providers, and durable medical equipment (DME) representatives. This clinic provides an opportunity for patients to share experiences and receive peer support, addressing common barriers to PAP use, such as mask discomfort, pressure settings, and anxiety. Preliminary evidence from a retrospective study at our institution [12] suggested that the SAM clinic model significantly improved PAP adherence. However, the potential incremental benefit of a fully multidisciplinary, group-based approach—incorporating real-time mask adjustments, interpersonal support, and individualized education in a single visit—has not been rigorously evaluated in a prospective manner.

Therefore, this randomized controlled trial examines whether a structured SAM clinic with multiple specialists, real-time interventions, and peer interaction can enhance PAP adherence and patient-reported outcomes beyond the levels reported in earlier studies [14–17]. We hypothesized that the SAM clinic intervention would improve PAP adherence and PROs compared to usual care and that results would inform future treatment approaches for managing OSA with PAP.

## Methods

### Study design

This study was designed as a randomized controlled trial (RCT) (NCT03835702) to evaluate the impact of a novel SAM group intervention on PAP adherence and PROs in patients with OSA. The trial aimed to provide robust evidence comparing the effectiveness of the SAM intervention with usual care.

### Participants

The study included adult participants aged 18 years and older who were newly diagnosed with OSA by either polysomnogram or home sleep apnea testing (apnea hypopnea index (AHI), respiratory disturbance index (RDI), Respiratory Event Index (REI) ≥ 5 events/hr) and had been prescribed PAP therapy. Key eligibility criteria included a new PAP setup within one month prior to enrollment and sub-optimal PAP adherence, defined as less than 70% usage and less than four hours of average daily usage, followed by sleep and non-sleep providers [18]. Exclusion criteria included central sleep apnea (where more than 50% of apneas were central), pregnancy, use of supplemental oxygen, inability to participate in group sessions if assigned to the SAM intervention (e.g., severe logistical or cognitive barriers), and inability to provide informed consent. Because randomization could assign patients to the SAM group, it was necessary to exclude those who could not meaningfully attend or engage in shared medical appointments, whether due to mobility, transportation, cognitive, or scheduling constraints.

**Fig. 1:**
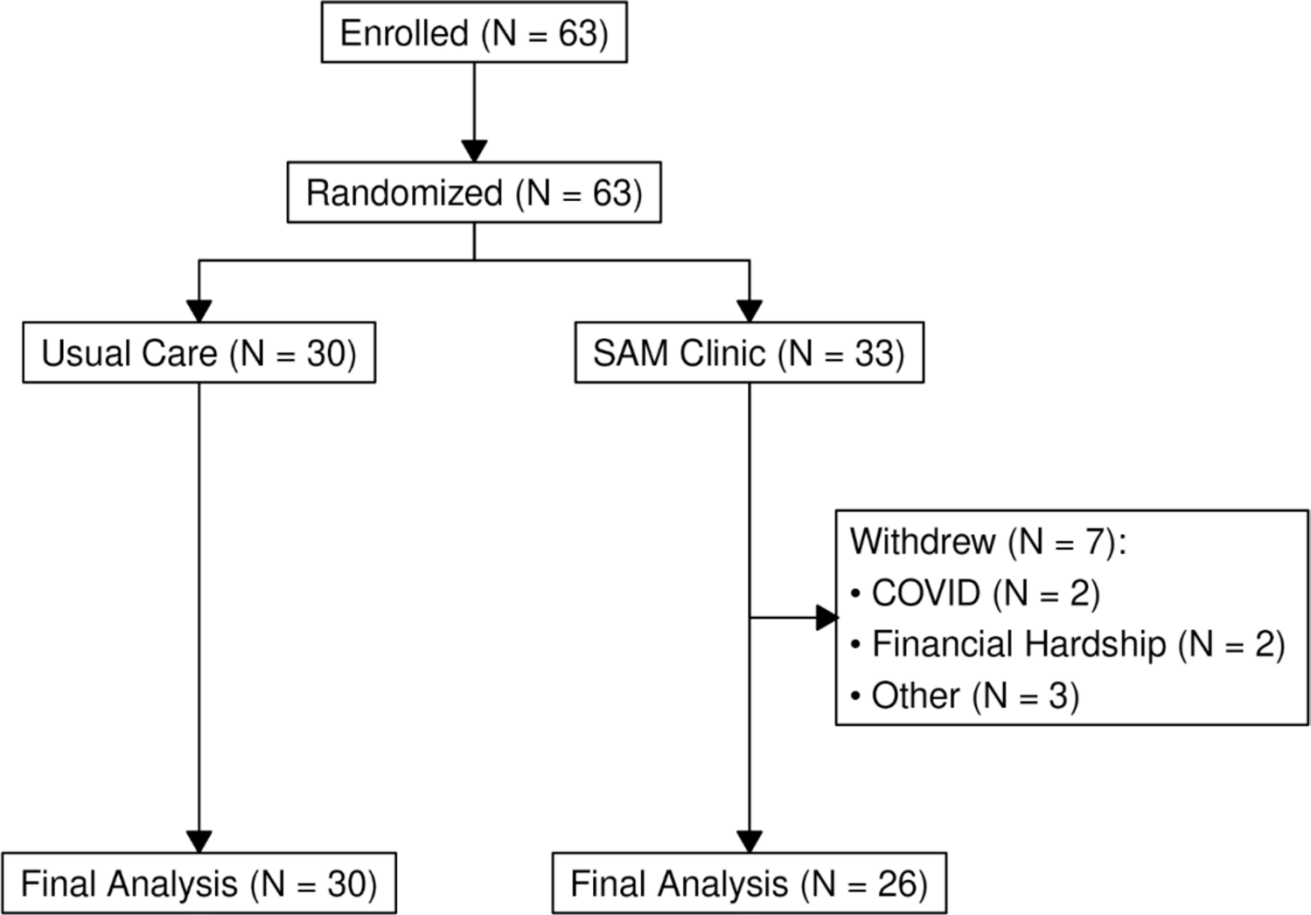
Participant recruitment and flow through the study

Participants were referred for PAP services through Cleveland Clinic Home Care by sleep providers. Potential participants were identified via electronic medical records and referrals from sleep specialists. They were contacted by research staff, who provided detailed information about the study and obtained informed consent. Following consent, participants were randomly assigned to either the SAM clinic intervention group or the usual care group using a computer-generated randomization scheme, ensuring equal distribution across groups. Figure 1 illustrates the flow of participants through the study.

Participants were included if they had OSA with an AHI > 5 events/hour and suboptimal PAP adherence, with stratification based on OSA severity (mild: AHI 5–14.9, moderate: AHI 15–29.9, and severe: AHI ≥ 30). While this reflects a critical subset of the OSA population, it may not fully represent the broader patient population initiating PAP therapy, including those with higher adherence. Recruitment relied on Cleveland Clinic DME data and included patients consenting to study participation, potentially introducing selection bias. Furthermore, while OSA is more prevalent in males, the higher proportion of females in this study may reflect healthcare engagement patterns or recruitment dynamics, warranting further investigation in future studies.

Sample size calculations determined that 166 participants (83 per group) would be needed to detect a 60-min difference in daily PAP usage, with an SD of 90 min, 80% power, and a significance level of 0.05. This estimate was based on retrospective data from our institution and similar interventions reported in the literature [12].

### Interventions

#### Sleep apnea management clinic

Participants in the SAM clinic group attended group-based sessions led by a multidisciplinary team, including sleep specialists, nurse practitioners, and DME representatives, following the organization's shared medical appointment format. This format includes a disclosure regarding confidentiality, which also applies to external DME representatives present at the sessions. These sessions focused on providing comprehensive education, troubleshooting, and support related to PAP therapy. Key topics included mask fitting and comfort, pressure settings, managing side effects such as nasal congestion and dry mouth, and addressing anxiety related to PAP use. The interactive and supportive environment of the SAM clinic aimed to enhance patient confidence and adherence to therapy. The SAM intervention was structured as a weekly recurring clinic, with individualized follow-up plans allowing for additional visits based on patient needs.

Each session involved a sleep medicine provider (sleep medicine physician or advanced practice provider) and usually one or two representatives from a DME company or a nurse proxy knowledgeable about PAP intricacies. About 6–10 patients were scheduled per session. Patients were instructed to arrive 15 min early to check in and fill out a questionnaire about their current machine and any issues they would like to discuss regarding therapy, including mask leak, skin irritation, improper mask fit, claustrophobia, nasal congestion, nose or mouth dryness, aerophagia, pressure intolerance, or other concerns. They were also instructed to bring their PAP machine and supplies to the visit.

The session began with a brief educational discussion about OSA and PAP therapy. Following this, the provider interacted with each patient personally to assess any individual issues interfering with PAP adherence. DME representatives were asked to intervene and help troubleshoot for individual patients during the visit, such as changing pressure settings and adjusting mask or headgear straps. Patients were encouraged to ask questions, share their experiences, and offer support as they felt comfortable. The provider formulated an individualized follow-up plan for each patient, which could include another SAM visit or a referral back to a sleep medicine specialist for more complicated issues. The SAM clinic was set up as a weekly recurring visit, with follow-up visits based on individual need, so each group varied from visit to visit. Throughout the manuscript, we occasionally use the terms “SAM clinic” and “SAM group” interchangeably to describe this multidisciplinary, group-based intervention.

Participants randomized to the SAM clinic were scheduled for an initial group session, with additional follow-up visits offered based on individual need and clinical judgment. Due to logistical constraints, including disruptions from the COVID-19 pandemic, all participants attended a single SAM clinic session during the study period, as recorded in the trial database. Descriptive statistics on session attendance indicate that, among the 26 participants in the SAM group, all had one documented SAM clinic visit, with a range of 1 to 1 sessions and a median of 1 session.

#### Usual care/Control arm

In contrast, participants in the usual care group received standard follow-up care from non-sleep specialists. Although these patients were not seen at a sleep center, they were followed by non-sleep providers for their PAP therapy, allowing for a comparison of the multidisciplinary SAM clinic approach to typical care outside of specialized sleep services. This care typically involved routine clinic visits after PAP setup and troubleshooting, but without the structured group support and education provided in the SAM clinic. This standard follow-up care focused on individual consultations, which, although comprehensive, lacked the synergistic benefits of the multidisciplinary and group-based approach of the SAM clinic. We refer to this non-sleep–specialist care pathway as the “usual care” or “control” arm throughout.

#### Sleep hygiene education

A research coordinator provided both arms with sleep hygiene education material at baseline that entailed regularization of sleep–wake schedule, stimulus control education, adequate amount of sleep, and a comfortable environment conducive to sleep by mail.

### Outcomes

The primary outcome of the study was PAP adherence, measured by the average hours of PAP use per night and the percentage of days with PAP use over a three-month period. Adherence data were collected using the PAP device’s built-in compliance monitoring system, which recorded usage statistics.

Secondary outcomes included changes in PROs, which are direct reports from patients about how they feel or function in relation to their health condition and its therapy, without interpretation by healthcare professionals. In this study, PROs focused on daytime sleepiness, quality of life, and depressive symptoms, and were administered as part of the research protocol through the REDCap platform. These were collected at pre-specified time points—within one to two weeks of baseline, at 1 month, and at 3 months—independent of clinical visits. The following PRO questionnaires were utilized: the Epworth Sleepiness Scale (ESS), an 8-item questionnaire widely used to measure subjective sleepiness, where an ESS score of > 10 indicates daytime sleepiness; [19] the Patient Health Questionnaire-9 (PHQ-9), a 9-item scale providing a psychometric measurement of depression, with a score of > 10 indicating moderate to severe depression [20]; the PROMIS Global Health, a 10-item questionnaire measuring patient-reported health status for physical, mental, and social well-being [21]; the PROMIS Sleep-Related Impairment, an 8-item questionnaire identifying sleep problems that affect overall sleep quality [22]; the PROMIS Fatigue, an 8-item questionnaire evaluating self-reported tiredness affecting daily activities and functioning [23]; and the PROMIS Anxiety, a 4-item questionnaire used to assess anxiety [24].

### Additional variables

Other variables collected included detailed sleep study-related variables such as the Apnea–Hypopnea Index (AHI), oxygen saturation levels, and types of PAP devices used. A detailed questionnaire specifically developed for this study aimed to capture common issues that patients might face while using PAP therapy, such as mask discomfort, nasal congestion, pressure intolerance, and other factors that could affect their adherence to the treatment. These barriers were assessed to understand the challenges patients encounter and to tailor interventions more effectively. Data from the questionnaire were collected at the same time points as the primary and secondary outcomes. We also collected baseline demographic and clinical variables (e.g., education level, tobacco/alcohol use, psychiatric disorders) for descriptive purposes to characterize our study population and assess balance between groups. Given the small sample size and the randomized design, additional covariates (e.g., education, smoking, neurological/psychiatric comorbidity) were not included in the primary models, as randomization was expected to balance these characteristics across arms.

### Statistical analysis

Categorical variables were described using frequencies and percentages, while continuous variables were described using means and standard deviations for normally distributed variables or medians and quartiles for non-normally distributed variables. Group balance was assessed using standardized differences.

Primary and secondary outcomes were evaluated using linear and logistic mixed-effects models. These models included time and group-by-time interactions as predictors and adjusted for the stratification variable of AHI group. To evaluate within-group changes, similar mixed models were fitted with group-specific intercepts, allowing for the evaluation of direct changes within each group.

The analysis was performed using SAS Software (version 9.4; Cary, NC). A significance level of 0.05 was assumed for all tests. Analyses used maximum likelihood estimation, which has been shown to provide similarly valid findings to multiple imputation for longitudinal analysis under the assumption that the data is missing at random [25].

We assessed model fit by examining residual plots and influence statistics (including Cook’s D) for outliers or influential observations. To evaluate the potential impact of missing data on the missing-at-random assumption, we compared mean changes among participants with missing data versus those with complete data at 1 and 3 months. Although our limited sample size reduces power to detect small violations, no substantial issues emerged that warranted further analysis.

Due to the unforeseen global COVID-19 pandemic, the study faced interruptions that led to incomplete data collection and potential impacts on participant engagement and adherence.

## Results

### Baseline characteristics

A total of 56 participants were included in the final analysis, with 26 randomized to the SAM clinic intervention group and 30 to the usual care group. The baseline characteristics of the participants are summarized in Table 1. The mean age of participants was 55.1 years (SD ± 14.5), with 42.9% male and 57.1% female. The groups were relatively well balanced, though empirically, differences in age, systolic blood pressure, and HbA1c levels were observed. Specifically, the usual care group had slightly higher systolic blood pressure and HbA1c than the SAM clinic group. Other demographic factors, including marital status, race, and education level, were similar between the groups.

**Table 1:** Baseline summarydemographics by group

	Overall (*N* = 56)		Randomized to SAM Clinic (*N* = 26)		Randomized to Usual Care (*N* = 30)		
Factor	N	Statistics	N	Statistics	N	Statistics	Standardized Difference
Demographics							
Age	56	55.1 ± 14.5	26	52.3 ± 12.6	30	57.6 ± 15.8	0.38
Marital status	56		26		30		0.58
Divorced		7(12.5)		3(11.5)		4(13.3)	
Legally separated		1(1.8)		1(3.8)		0(0.0)	
Married		24(42.9)		13(50.0)		11(36.7)	
Single		21(37.5)		7(26.9)		14(46.7)	
Unknown		1(1.8)		1(3.8)		0(0.0)	
Widowed		2(3.6)		1(3.8)		1(3.3)	
Gender at birth	56		26		30		0.021
Male		24(42.9)		11(42.3)		13(43.3)	
Female		32(57.1)		15(57.7)		17(56.7)	
Patient Race	56		26		30		0.32
Black or African American		20(35.7)		10(38.5)		10(33.3)	
White		35(62.5)		15(57.7)		20(66.7)	
Other		1(1.8)		1(3.8)		0(0.0)	
Ethnicity	56		26		30		0.26
Not Hispanic or Latino/a		55(98.2)		26(100.0)		29(96.7)	
Hispanic or Latino/a		1(1.8)		0(0.0)		1(3.3)	
Height (in cm)	56	168.9 ± 10.9	26	168.9 ± 11.0	30	169.0 ± 11.0	0.007
Weight (in kg)	56	97.4 ± 25.5	26	97.2 ± 28.6	30	97.5 ± 23.0	0.015
BMI	56	34.2 ± 8.6	26	34.0 ± 9.3	30	34.3 ± 8.1	0.031
Systolic BP	56	126.6 ± 18.1	26	122.0 ± 16.8	30	130.7 ± 18.4	0.50
Diastolic BP	56	75.7 ± 11.9	26	74.8 ± 10.8	30	76.5 ± 12.9	0.15
Hemoglobin A1c	44	6.1 ± 1.05	20	5.9 ± 0.73	24	6.2 ± 1.3	0.37
Baseline AHI Group	56		26		30		0.086
Baseline ahi 5–14.9		19(33.9)		9(34.6)		10(33.3)	
Baseline ahi 15–29		19(33.9)		9(34.6)		10(33.3)	
Baseline ahi ≥ 30		18(32.1)		8(30.8)		10(33.3)	
What is the highest degree or level of school you have completed?	55		26		29		0.55
No schooling completed		1(1.8)		0(0.0)		1(3.4)	
Some high school, no diploma		3(5.5)		2(7.7)		1(3.4)	
High school graduate or equivalent (GED)		5(9.1)		2(7.7)		3(10.3)	
Some college credit, no degree		15(27.3)		6(23.1)		9(31.0)	
Associate's degree		13(23.6)		7(26.9)		6(20.7)	
Bachelor's degree		10(18.2)		5(19.2)		5(17.2)	
Master's degree		6(10.9)		2(7.7)		4(13.8)	
Professional degree		1(1.8)		1(3.8)		0(0.0)	
Doctorate degree		1(1.8)		1(3.8)		0(0.0)	
Has the participant ever used/smoked tobacco?	56	34(60.7)	26	17(65.4)	30	17(56.7)	0.18
Specify tobacco use:	34		17		17		0.15
Past		27(79.4)		14(82.4)		13(76.5)	
Current		7(20.6)		3(17.6)		4(23.5)	
If current, average number of packs per week: (please use exact number, range not acceptable)	6	2.3[1.00,4.0]	2	2.5[1.00,4.0]	4	2.3[1.00,5.3]	0.25
Does the participant drink alcohol?	56	33(58.9)	26	16(61.5)	30	17(56.7)	0.099
If they drink alcohol, average number of glasses per week: (Please use exact number, range not acceptable)	19	2.0[1.00,6.0]	7	2.0[1.00,2.5]	12	2.3[1.5,6.0]	0.64
Statistics presented as Mean ± SD, Median[Q1, Q3] or N (column %)							

Medical history and medication use at baseline are presented in Table 2. Participants in both groups had a high prevalence of cardiovascular diseases, with 75% reporting such conditions. The usual care group had a higher percentage of participants using more than 15 medications compared to the SAM clinic group. Sleep study characteristics at baseline, shown in Table 3, indicated that both groups were similar in terms of AHI, oxygen saturation levels, and types of PAP devices used. Most participants used auto-CPAP devices, and common barriers to PAP use included mask discomfort and nasal congestion.

**Table 2:** Baseline summary—medical history and medications by group

	Overall (*N* = 56)		Randomized to SAM Clinic (*N* = 26)		Randomized to Usual Care (*N* = 30)		
Factor	N	Statistics	N	Statistics	N	Statistics	Standardized Difference
Any systemic disease							
Sleep Disorders other than OSA	56	19(33.9)	26	10(38.5)	30	9(30.0)	0.18
HEENT other than OSA	56	12(21.4)	26	4(15.4)	30	8(26.7)	0.28
Cardiovascular	56	42(75.0)	26	18(69.2)	30	24(80.0)	0.25
Respiratory	56	15(26.8)	26	5(19.2)	30	10(33.3)	0.32
COPD	56	2(3.6)	26	1(3.8)	30	1(3.3)	0.028
Medication use							
Does the participant currently use any (prescription) medications?	56	55(98.2)	26	25(96.2)	30	30(100.0)	0.28
How many? (number of different types of medications)	56		26		30		0.84
0–5		12(21.4)		6(23.1)		6(20.0)	
6–10		19(33.9)		13(50.0)		6(20.0)	
11–15		13(23.2)		5(19.2)		8(26.7)	
Over 15		12(21.4)		2(7.7)		10(33.3)	
Sleep Disorders							
Restless leg syndrome	56	3(5.4)	26	2(7.7)	30	1(3.3)	–
Nightmares/Night terrors	56	0(0.0)	26	0(0.0)	30	0(0.0)	–
Insomnia	56	13(23.2)	26	6(23.1)	30	7(23.3)	–
Narcolepsy	56	0(0.0)	26	0(0.0)	30	0(0.0)	–
Sleepwalking	56	0(0.0)	26	0(0.0)	30	0(0.0)	–
Other	56	8(14.3)	26	5(19.2)	30	3(10.0)	–
None	56	37(66.1)	26	16(61.5)	30	21(70.0)	–
Cardiovascular							
Hyperlipidemia	56	27(48.2)	26	11(42.3)	30	16(53.3)	–
Elevated blood pressure	56	2(3.6)	26	1(3.8)	30	1(3.3)	–
Hypertension	56	32(57.1)	26	13(50.0)	30	19(63.3)	–
Myocardial infarction	56	2(3.6)	26	1(3.8)	30	1(3.3)	–
Congestive heart failure	56	5(8.9)	26	3(11.5)	30	2(6.7)	–
Heart disease	56	0(0.0)	26	0(0.0)	30	0(0.0)	–
Coronary artery disease	56	3(5.4)	26	2(7.7)	30	1(3.3)	–
Cancer	56	0(0.0)	26	0(0.0)	30	0(0.0)	–
Atrial fibrillation	56	3(5.4)	26	0(0.0)	30	3(10.0)	–
Other	56	19(33.9)	26	9(34.6)	30	10(33.3)	–
Respiratory							
Chronic obstructive pulmonary disease (COPD)	56	4(7.1)	26	3(11.5)	30	1(3.3)	–
Cystic fibrosis	56	0(0.0)	26	0(0.0)	30	0(0.0)	–
Asthma	56	9(16.1)	26	3(11.5)	30	6(20.0)	–
COPD Types							
Emphysema	56	2(3.6)	26	1(3.8)	30	1(3.3)	–
Chronic bronchitis	56	0(0.0)	26	0(0.0)	30	0(0.0)	–
Neurology							
Migraine	56	5(8.9)	26	3(11.5)	30	2(6.7)	–
Psychiatric							
Attention deficit disorder/Attention deficit hyperactivity disorder (ADD/ADHD)	56	1(1.8)	26	0(0.0)	30	1(3.3)	–
Depression	56	21(37.5)	26	10(38.5)	30	11(36.7)	–
Anxiety	56	16(28.6)	26	5(19.2)	30	11(36.7)	–
Post-traumatic stress disorder (PTSD)	56	4(7.1)	26	2(7.7)	30	2(6.7)	–
Seasonal affective disorder	56	0(0.0)	26	0(0.0)	30	0(0.0)	–
Other	56	8(14.3)	26	4(15.4)	30	4(13.3)	–
None	56	28(50.0)	26	13(50.0)	30	15(50.0)	–
Statistics presented as Median[Q1, Q3] or N (column %)

**Table 3:** Baseline summary—sleep report by group

	Overall (*N* = 56)		Randomized to SAM Clinic (*N* = 26)		Randomized to Usual Care (*N* = 30)		
Factor	N	Statistics	N	Statistics	N	Statistics	Standardized Difference
ESS total score on most recent sleep study	55	10.0[5.0,13.0]	25	10.0[7.0,13.0]	30	10.0[5.0,13.0]	0.15
Sleep study type	56		26		30		0.60
PSG		22(39.3)		14(53.8)		8(26.7)	
HSAT		8(14.3)		2(7.7)		6(20.0)	
Split night		26(46.4)		10(38.5)		16(53.3)	
Total recording time (in min)	56	401.5 ± 56.5	26	397.5 ± 58.8	30	404.9 ± 55.3	0.13
Sleep time in supine position in minutes	52	93.1[36.2,165.3]	24	101.8[44.7,191.5]	28	85.3[36.2,165.3]	0.13
AHI (events/hr)	49	22.8[9.3,39.6]	25	20.1[8.4,33.3]	24	23.8[10.5,50.7]	0.40
RDI (events/hr)	17	15.3[8.4,21.8]	12	16.3[8.3,23.2]	5	11.1[10.3,21.8]	0.24
REI (events/hr)	7	8.3[6.1,24.3]	1	48.8[48.8,48.8]	6	7.3[6.1,19.5]	N/A
Number of apneas	55	7.0[1.00,14.0]	26	7.0[0.00,13.0]	29	5.0[1.00,14.0]	0.028
Number of hypopneas	56	33.0[21.0,58.0]	26	29.5[19.0,77.4]	30	34.5[24.0,49.0]	0.28
Central apnea index (CAI) (events/hr)	26	0.00[0.00,1.00]	10	0.00[0.00,1.00]	16	0.00[0.00,0.75]	0.083
Minimal measured SaO2 (%)	56	84.5[81.0,88.5]	26	84.5[81.0,89.0]	30	84.5[81.0,88.0]	0.12
Mean SaO2 (%)	56	93.5[91.0,95.0]	26	94.0[91.0,95.0]	30	93.0[91.0,95.0]	0.26
Total time SaO2 < 90% (min)	51	3.5[0.20,17.9]	24	0.70[0.20,16.3]	27	5.9[1.2,25.9]	0.23
Percentage of total time SaO2 < 90% (min)	51	1.6[0.20,20.6]	24	0.65[0.10,17.4]	27	2.7[1.00,20.6]	0.33
Percentage of total time SaO2 < 88% (min)	48	0.95[0.00,12.7]	22	0.30[0.00,7.6]	26	1.1[0.70,14.5]	0.47
Total time SaO2 < 88% (min)	47	1.1[0.10,14.8]	21	0.30[0.00,7.0]	26	3.5[0.60,15.5]	0.19
PAP Characteristics							
Type of PAP	55		25		30		0.33
CPAP		8(14.5)		5(20.0)		3(10.0)	
BiPAP		2(3.6)		1(4.0)		1(3.3)	
Auto CPAP		39(70.9)		17(68.0)		22(73.3)	
Auto BiPAP		6(10.9)		2(8.0)		4(13.3)	
What kind of mask do you have?: Nasal mask	56	17(30.4)	26	8(30.8)	30	9(30.0)	0.017
What kind of mask do you have?: Full face mask	56	18(32.1)	26	7(26.9)	30	11(36.7)	0.21
What kind of mask do you have?: Nasal pillows	56	18(32.1)	26	10(38.5)	30	8(26.7)	0.25
What kind of mask do you have?: Hybrid mask	56	4(7.1)	26	2(7.7)	30	2(6.7)	0.040
Are you using the humidifier?	55	48(87.3)	26	22(84.6)	29	26(89.7)	0.15
Do you feel you are benefitting from PAP therapy?	55	37(67.3)	26	14(53.8)	29	23(79.3)	0.56
Do you wear a chin strap?	55		26		29		0.29
No		43(78.2)		21(80.8)		22(75.9)	
Yes		9(16.4)		3(11.5)		6(20.7)	
Sometimes		3(5.5)		2(7.7)		1(3.4)	
Is your family supportive of your efforts to treat your sleep apnea?	55		26		29		0.37
No		1(1.8)		1(3.8)		0(0.0)	
Yes		51(92.7)		23(88.5)		28(96.6)	
Other		3(5.5)		2(7.7)		1(3.4)	
PAP Barriers							
Do you have difficulty tolerating the pressure?	56	16(28.6)	26	9(34.6)	30	7(23.3)	0.25
Is the pressure:	16		9		7		0.10
Too low		5(31.3)		3(33.3)		2(28.6)	
Too high		11(68.8)		6(66.7)		5(71.4)	
Do you have air swallowing or feel bloated?	55	15(27.3)	26	6(23.1)	29	9(31.0)	0.18
Do you have leaking around the mask?	55	26(47.3)	26	14(53.8)	29	12(41.4)	0.25
Do you have skin irritation related to the mask?	56	12(21.4)	26	8(30.8)	30	4(13.3)	0.43
Do you have mask fitting problems?	56	22(39.3)	26	11(42.3)	30	11(36.7)	0.12
Do you have mask claustrophobia?	56	17(30.4)	26	7(26.9)	30	10(33.3)	0.14
Do you have nasal congestion?	55	33(60.0)	26	16(61.5)	29	17(58.6)	0.060
Do you have nose/mouth dryness?	56	39(69.6)	26	15(57.7)	30	24(80.0)	0.50
Statistics presented as Median[Q1, Q3] or N (column %)							

### PAP adherence

The primary outcome of PAP adherence was measured by average hours of PAP use and the percentage of days with PAP use over three months.

At one month, participants in the SAM clinic group showed a trend towards higher PAP adherence, with an average usage of 209.3 min compared to 177.3 min in the usual care group. However, this difference was not statistically significant (Table 4). By three months, the average usage in the SAM clinic group was 193.2 min compared to 147.5 min in the usual care group, approaching statistical significance (*p* = 0.093) as shown in Table 4. A supplementary forest plot (Supplementary Fig. 1) illustrates the between-group differences in key outcomes at both 1 month and 3 months, with 95% confidence intervals.

**Table 4:** Continuous survey comparisons at 1 month and 3 months

Response	SAM Estimate (95% CI)	Usual Care Estimate (95% CI)	Difference Estimate (95% CI)	*P*-value
1 month				
ESS	10.4(8.1,12.7)	8.7(7.1,10.4)	1.7(−0.12,3.5)	0.068
PHQ-9	8.6(6.2,11.0)	8.1(6.3,9.8)	0.56(−1.2,2.3)	0.53
PROMIS Physical	41.4(37.7,45.2)	41.7(38.9,44.4)	−0.26(−3.0,2.5)	0.85
PROMIS Mental	42.8(38.7,46.8)	42.0(39.0,45.0)	0.75(−2.1,3.6)	0.60
PROMIS Anxiety	54.6(49.9,59.4)	54.2(50.8,57.5)	0.46(−3.2,4.1)	0.80
PROMIS Fatigue	58.7(54.3,63.1)	57.9(54.8,61.1)	0.79(−2.6,4.1)	0.64
PROMIS SRI	52.2(48.3,56.1)	52.7(49.9,55.5)	−0.50(−3.5,2.5)	0.74
Average Use (minutes)	209.3(155.6,263.0)	177.3(139.7,214.9)	32.0(−12.5,76.5)	0.16
3 months				
ESS	10.0(7.7,12.3)	9.3(7.7,11.0)	0.68(−1.5,2.9)	0.55
PHQ-9	8.2(5.8,10.6)	8.1(6.4,9.9)	0.07(−2.2,2.3)	0.95
PROMIS Physical	42.0(38.2,45.8)	42.3(39.6,45.0)	−0.31(−3.8,3.2)	0.86
PROMIS Mental	43.7(39.7,47.8)	45.9(42.9,48.9)	−2.1(−5.9,1.6)	0.26
PROMIS Anxiety	54.9(50.1,59.6)	51.7(48.4,55.0)	3.2(−1.4,7.7)	0.17
PROMIS Fatigue	57.2(52.8,61.5)	56.4(53.3,59.5)	0.73(−3.4,4.9)	0.73
PROMIS SRI	51.4(47.4,55.3)	51.2(48.4,53.9)	0.20(−3.6,4.0)	0.92
Average Use (minutes)	193.2(139.2,247.2)	147.5(110.3,184.7)	45.7(−7.8,99.1)	0.093^a^

^a^The *p*-values represent the mean difference between the SAM and usual care groups at each time point, as estimated from the mixed-effects model adjusting for AHI group. A group-by-time interaction term was included in the model to estimate time-specific differences in a single framework, but the corresponding interaction *p*-value is not reported here because our primary focus is the mean difference at each time point

### Patient-reported outcomes (PROs)

PROs were assessed using the ESS, PHQ-9, and PROMIS Global Health scales. Baseline scores for ESS, PHQ-9, and PROMIS instruments, as well as average PAP usage minutes, are provided in Supplement Table 1.

- Epworth Sleepiness Scale (ESS): At one month, the ESS scores in the SAM clinic group showed minimal change (−0.05, 95% CI: −1.5 to 1.3, *p* = 0.94), while the Usual Care group demonstrated a significant improvement (−2.4, 95% CI: −3.7 to −1.1, *p* < 0.001). Similar patterns were observed at 3 months, with the SAM group showing a small, non-significant change (−0.65, 95% CI: −2.5 to 1.2, *p* = 0.48), and the Usual Care group showing significant improvement (−2.5, 95% CI: −4.2 to −0.83, *p* = 0.004). (Table 5).
- Patient Health Questionnaire-9 (PHQ-9): Both groups exhibited significant reductions in PHQ-9 scores at one month (SAM: −1.5, 95% CI: −2.9 to −0.18; *p* = 0.026; Usual Care: −2.1, 95% CI: −3.4 to −0.90; *p* < 0.001) and three months (SAM: −2.2, 95% CI: −3.9 to −0.37; *p* = 0.019; Usual Care: −2.3, 95% CI: −4.0 to −0.67; *p* = 0.006), reflecting decreased depressive symptoms. (Tables 5).
- PROMIS Global Health Scales: PROMIS scores for physical health, mental health, anxiety, and fatigue did not show significant differences between the SAM and usual care groups at either one or three months. However, within-group analyses revealed significant improvements in Sleep-Related Impairment (SRI) scores at 1 month for both groups (SAM: −2.6, 95% CI: −5.0 to −0.20; *p* = 0.034; Usual Care: −2.3, 95% CI: −4.5 to −0.06; *p* = 0.044). By three months, the improvement in SRI was sustained in the usual care group (Usual Care: −3.5, 95% CI: −6.4 to −0.60; *p* = 0.019), though the change in the SAM group was no longer statistically significant (SAM: −3.0, 95% CI: −6.2 to 0.11; *p* = 0.059).
- PROMIS Anxiety and Fatigue Scales: Both the SAM and usual care groups showed small and non-significant improvements in PROMIS Anxiety and Fatigue scores at both one and three months. For example, the PROMIS Anxiety scores decreased slightly within the SAM group at one month by −1.07 points (95% CI: −3.91 to 1.8; *p* = 0.46) and within the usual care group by −1.8 points (95% CI: −4.50 to 0.83; *p* = 0.18).

**Table 5:** Continuous survey within group changes at 1 month and 3 months

	SAM		Usual Care	
Response	Estimate (95% CI)	*P*-value	Estimate (95% CI)	*P*-value
1 month				
ESS	−0.05(−1.5,1.3)	0.94	−2.4(−3.7,−1.1)	** < *0.001***
PHQ-9	−1.5(−2.9,−0.18)	***0.026***	−2.1(−3.4,−0.90)	** < *0.001***
PROMIS Physical	−0.13(−2.2,2.0)	0.90	0.39(−1.5,2.3)	0.69
PROMIS Mental	0.49(−1.7,2.7)	0.66	−0.37(−2.4,1.7)	0.72
PROMIS Anxiety	−1.07(−3.9,1.8)	0.46	−1.8(−4.5,0.83)	0.18
PROMIS Fatigue	−0.54(−3.2,2.1)	0.68	−1.10(−3.5,1.3)	0.36
PROMIS SRI	−2.6(−5.0,−0.20)	***0.034***	−2.3(−4.5,−0.06)	***0.044***
Average Use (minutes)	20.5(−15.2,56.1)	0.26	−9.3(−43.0,24.4)	0.59
3 months				
ESS	−0.65(−2.5,1.2)	0.48	−2.5(−4.2,−0.83)	***0.004***
PHQ-9	−2.2(−3.9,−0.37)	***0.019***	−2.3(−4.0,−0.67)	***0.006***
PROMIS Physical	0.31(−2.5,3.1)	0.83	1.09(−1.5,3.7)	0.41
PROMIS Mental	0.07(−2.9,3.0)	0.96	2.0(−0.74,4.8)	0.15
PROMIS Anxiety	0.65(−3.1,4.4)	0.73	−3.0(−6.5,0.46)	0.088
PROMIS Fatigue	−2.2(−5.6,1.2)	0.20	−2.6(−5.7,0.63)	0.11
PROMIS SRI	−3.0(−6.2,0.11)	0.059	−3.5(−6.4,−0.60)	***0.019***
Average Use (min	10.6(−35.6,56.9)	0.65	−31.7(−75.1,11.7)	0.15^a^

^a^This table presents within-group changes from baseline to each follow-up. Because the primary hypothesis concerns between-group differences (see Table 4), the interaction *p*-value is not provided here. The mixed-effects models do include an interaction term to handle repeated measures, but the statistics shown reflect within-group changes only

### Barriers to PAP adherence

The tertiary outcome focused on barriers to PAP adherence.

- Between-Group Comparisons: At one month, the SAM clinic group showed a trend towards lower rates of pressure intolerance compared to the usual care group, but this did not reach statistical significance (*p* = 0.087) By three months, the SAM clinic group had lower rates of nose/mouth dryness compared to the usual care group, though this difference was also not statistically significant (*p* = 0.097) (Table 6).
- Within-Group Analyses: Within-group analyses indicated that the usual care group experienced trends towards a reduction in mask claustrophobia and nasal congestion over time, while the SAM clinic group showed trends towards a reduction in skin irritation, although these findings were not statistically significant (Tables 7).

**Table 6:** Barrier comparisons at 1 month and 3 months

Response	SAM Estimate (95% CI)	Usual Care Estimate (95% CI)	Odds Ratio Estimate (95% CI)	*P*-value
1 month				
Pressure Toleration	0.08(0.02,0.30)	0.27(0.11,0.53)	0.25(0.05,1.2)	0.087
Air Swallowing	0.14(0.03,0.43)	0.14(0.04,0.37)	0.95(0.18,5.0)	0.95
Leaking	0.57(0.24,0.85)	0.68(0.39,0.87)	0.64(0.15,2.8)	0.55
Skin Irritation	0.14(0.03,0.46)	0.23(0.08,0.53)	0.55(0.11,2.7)	0.46
Mask fitting	0.29(0.08,0.65)	0.51(0.23,0.77)	0.39(0.09,1.8)	0.22
Mask Claustrophobia	0.34(0.10,0.72)	0.29(0.10,0.59)	1.3(0.26,6.6)	0.74
Nasal Congestion	0.71(0.31,0.93)	0.64(0.32,0.87)	1.4(0.25,7.5)	0.71
Nose/Mouth Dryness	0.44(0.13,0.81)	0.66(0.32,0.89)	0.41(0.08,2.2)	0.29
3 months				
Pressure Toleration	0.20(0.06,0.50)	0.28(0.11,0.56)	0.64(0.16,2.5)	0.52
Air Swallowing	0.24(0.07,0.59)	0.33(0.13,0.63)	0.65(0.14,2.9)	0.57
Leaking	0.42(0.15,0.75)	0.18(0.06,0.44)	3.2(0.69,14.7)	0.14
Skin Irritation	0.10(0.02,0.35)	0.08(0.02,0.28)	1.2(0.21,7.0)	0.83
Mask fitting	0.14(0.03,0.42)	0.16(0.05,0.41)	0.83(0.17,4.2)	0.82
Mask Claustrophobia	0.29(0.08,0.67)	0.25(0.08,0.56)	1.2(0.23,6.2)	0.83
Nasal Congestion	0.71(0.31,0.93)	0.63(0.32,0.86)	1.4(0.26,7.7)	0.69
Nose/Mouth Dryness	0.61(0.24,0.88)	0.89(0.60,0.98)	0.20(0.03,1.3)	0.097

**Table 7:** Categorical barrier within group changes at 1 month and 3 months

	SAM		Usual Care	
Response	Odds Ratio Estimate (95% CI)	*P*-value	Odds Ratio Estimate (95% CI)	*P*-value
1 month				
Pressure Toleration	0.29(0.07,1.3)	0.099	1.8(0.52,6.6)	0.34
Air Swallowing	0.77(0.17,3.5)	0.73	0.56(0.15,2.1)	0.39
Leaking	0.68(0.18,2.5)	0.56	1.7(0.50,5.6)	0.41
Skin Irritation	0.48(0.11,2.0)	0.32	2.2(0.51,9.7)	0.28
Mask fitting	0.67(0.18,2.5)	0.55	2.1(0.62,7.3)	0.23
Mask Claustrophobia	1.8(0.41,7.6)	0.44	1.00(0.27,3.8)	0.99
Nasal Congestion	1.07(0.23,5.0)	0.93	0.84(0.23,3.0)	0.78
Nose/Mouth Dryness	0.95(0.23,3.9)	0.95	1.00(0.23,4.3)	0.99
3 months				
Pressure Toleration	1.2(0.35,4.2)	0.75	3.1(0.89,10.9)	0.074
Air Swallowing	1.3(0.31,5.4)	0.72	1.3(0.38,4.7)	0.64
Leaking	0.81(0.22,3.0)	0.74	0.43(0.12,1.5)	0.20
Skin Irritation	0.47(0.11,2.0)	0.29	1.00(0.20,5.0)	0.99
Mask fitting	0.39(0.10,1.6)	0.18	0.60(0.16,2.2)	0.44
Mask Claustrophobia	1.3(0.31,5.6)	0.71	0.82(0.21,3.2)	0.77
Nasal Congestion	1.07(0.23,5.0)	0.93	0.84(0.23,3.0)	0.78
Nose/Mouth Dryness	1.3(0.31,5.3)	0.72	2.8(0.50,15.4)	0.24

## Discussion

This randomized controlled trial evaluated the impact of a SAM group intervention on PAP adherence and PROs in individuals with OSA. The study was terminated prematurely; therefore, an adequate determination of the efficacy of the SAM clinic versus usual care could not be made. The study originally planned to enroll 166 participants (83 per group), but only 56 participants (approximately 34% of the target) were included in the final analysis, with 26 randomized to the SAM clinic intervention group and 30 to the usual care group. However, the findings suggest that the SAM group intervention has the potential to provide a valid and efficient process for care in individuals with OSA, though further research is needed to confirm its impact on PAP adherence and patient-reported outcomes. While the differences in adherence and PROs between the SAM clinic and usual care groups did not reach statistical significance, trends observed over the three months indicate potential clinical benefits associated with the SAM intervention. Although there were trends toward improved adherence in the SAM group, the PROs (ESS, PHQ-9, and PROMIS measures) did not show significant between-group differences. One possible explanation is the small sample size and the resulting limited power to detect differences in subjective outcomes. Additionally, the three-month follow-up period may have been insufficient for participants to experience or report meaningful changes in well-being. While a 3-month window is often tied to DME reimbursement requirements in certain healthcare systems, larger clinical trials [26] and extensive real-world datasets [14, 27] suggest that the most significant drop in PAP use commonly occurs around or after 3 months. Future investigations with extended follow-up periods of 6 to 12 months may therefore be more informative for assessing the sustained efficacy of the SAM model. It is also possible that the SAM clinic’s focus on immediate PAP troubleshooting and adherence support, while valuable for usage, may not fully address the multifaceted behavioral or psychosocial factors captured by these PRO instruments. Additionally, both groups showed significant improvements in depressive symptoms and daytime sleepiness, although the usual care group demonstrated a more pronounced reduction in ESS scores.

Our previous retrospective findings from our institution, showed significant improvements in PAP adherence following the implementation of the SAM clinic model [12]. The retrospective study demonstrated that PAP adherence rates improved from 46% at baseline to 66% at follow-up, with average PAP usage increasing by 1.2 h. This RCT showed trends towards higher PAP adherence in the SAM clinic group, reinforcing the effectiveness of the SAM clinic model in enhancing adherence to PAP therapy [12]. Additionally, studies by Likar et al. and Lettieri et al. have demonstrated the benefits of group education sessions [13, 28]. Likar et al. found that a 2-h group CPAP clinic significantly increased nightly CPAP use from 5.2 to 6.3 h, an improvement sustained over 605 days​(likar). Similarly, Lettieri et al. reported that group educational programs resulted in better CPAP adherence compared to individual counseling, with patients using CPAP on more nights (67.2% vs. 62.1%) and for more hours per night (4.3 vs. 3.7 h) [12]. Our study differs from those by Likar et al. and Lettieri et al. in several key aspects, providing additional insights and evidence. Unlike the retrospective chart reviews and performance improvement initiatives used by Likar et al. and Lettieri et al., our study was an RCT, which provides a higher level of evidence by minimizing potential biases and confounding variables. This RCT design allows us to more confidently attribute observed outcomes to the SAM intervention rather than to external factors. Furthermore, the structure of the group clinics in our study was specifically designed to focus not only on education and equipment support but also on behavioral strategies tailored to improve long-term adherence and PROs. While Likar et al. emphasized equipment monitoring and symptom treatment in their CPAP clinics, our SAM group included personalized goal-setting, problem-solving strategies, and peer support dynamics, which might have contributed to the nuanced differences in outcomes. Additionally, our study evaluated a wider range of outcomes, including PROs like the PHQ-9 and PROMIS Global Health scales. By doing so, we not only assessed CPAP adherence but also captured the broader impact of the intervention on patients' overall health and well-being, which previous studies did not explore in depth. Lastly, we conducted follow-ups at multiple time points (1 month and 3 months), providing a more comprehensive view of how CPAP adherence and PROs evolve over time. Although these repeated assessments are a strength, we observed that the intervention’s impact on PAP usage appeared more pronounced at 1 month (209.3 min) compared to 3 months (193.2 min). Participants randomized to the SAM clinic were scheduled for an initial group session, with additional follow-up visits offered based on individual need and clinical judgment. Due to logistical constraints, including disruptions from the COVID-19 pandemic, all participants attended a single SAM clinic session during the study period, as recorded in the trial database. Descriptive statistics on session attendance indicate that, among the 26 participants in the SAM group, all had one documented SAM clinic visit, with a range of 1 to 1 sessions and a median of 1 session. This single-session exposure, typically occurring within the first month, likely contributed to the initial boost in adherence, which diminished over time without further reinforcement. Understanding whether additional “booster” sessions or extended follow-up can sustain improved adherence remains an important area for future research. Together, these elements highlight how our study not only builds on existing literature but also offers a more robust and comprehensive evaluation of group education’s impact on CPAP adherence and overall patient outcomes.

The success of the SAM clinic model can be attributed to its multidisciplinary approach and the provision of peer support. The SAM group sessions, led by sleep specialists, advanced practice providers, and DME representatives, provided a comprehensive platform for education, troubleshooting, and support. The opportunity for patients to share experiences and receive peer encouragement likely contributed to the observed adherence improvements, aligning with findings from other studies that have shown the effectiveness of group education and peer support in enhancing treatment adherence across various medical conditions. This distinguishes the SAM model from previously studied interventions such as telemedicine-based education (which can enhance CPAP usage but may limit real-time mask adjustments) [14, 15] or educational video tools (effective for CPAP acceptance but often lacking ongoing support) [1, 16]. Likewise, targeted cognitive behavioral therapy and supportive follow-up can improve adherence, [17] yet these approaches typically address a single behavioral aspect rather than combining live troubleshooting, peer interaction, and immediate equipment adjustments under one roof. By incorporating real-time DME support, immediate mask refitting, and shared experiences among participants, SAM has the advantage of addressing multiple adherence barriers in a single, structured forum. The clinic’s unique design, characterized by regular, billable clinical visits, maximizes accessibility and allows for prompt problem-solving. However, this model may require more staffing resources, scheduling coordination, and patient availability for group sessions, posing logistical challenges. We recognize that many usual-care pathways also involve DME representatives and respiratory therapists to address equipment tolerance, mask fit, and pressure settings. However, our SAM approach sought to ensure these specialists were present in real time at a shared medical appointment, enabling immediate troubleshooting and peer feedback. Although this may not be entirely unique to our setting, the goal was to formalize and standardize such interactions within a group-based, multidisciplinary clinic.

While many sleep centers do offer individualized troubleshooting for PAP therapy, the SAM clinic model is unique in that it provides a structured, scheduled group session—under a shared medical appointment format—where patients can receive real-time equipment adjustments, professional advice from sleep medicine providers and DME specialists, and benefit from peer support and interaction. This differs from standard visits or telemonitoring approaches, which typically involve one-on-one appointments or remote follow-up without the synergistic group component. Furthermore, we integrated behavioral counseling, mask fit services, and immediate device recalibration in a single session for multiple patients simultaneously, aiming to enhance adherence through both professional guidance and the supportive dynamics of a group setting.

The study’s sample size was substantially below the calculated target of 166 participants due to constraints related to the COVID-19 pandemic, which necessitated premature termination of recruitment. This underpowering likely affected the ability to detect statistically significant differences in PAP adherence and patient-reported outcomes. Moreover, the small sample size may limit the generalizability of our findings to broader populations, as subgroup analyses and nuanced comparisons were not feasible. Additionally, pandemic-related disruptions (e.g., delayed clinical visits, reduced staffing for SAM sessions, and increased patient hesitancy for in-person appointments) may have affected adherence, participation rates, and the consistency of data collection. Some participants faced logistical challenges or health concerns that potentially reduced their engagement with the study, introducing further limitations in assessing the true effect of the intervention. Recruitment was conducted via electronic medical records, and participants volunteered upon learning about the study; this self-selection may introduce bias, as individuals who are more motivated or health-conscious could be overrepresented, potentially inflating adherence and engagement outcomes. Although a higher proportion of female participants was enrolled, randomization and baseline balance suggest no systematic bias in study outcomes. It is possible that women’s higher healthcare engagement contributed to this overrepresentation, but further research is needed to determine whether gender differences in OSA presentation or treatment adherence affect results.

The follow-up period of three months may also be insufficient to capture long-term adherence trends. Furthermore, reliance on self-reported measures for some PROs may introduce bias, although the use of validated instruments helps mitigate this issue. Another potential source of bias involves participants’ ability and motivation to attend SAM sessions, as logistical or personal barriers may have led some individuals to withdraw from the intervention arm, potentially affecting the observed outcomes.

Future studies should aim to include larger sample sizes and longer follow-up periods to confirm our findings and explore the long-term impact of the SAM intervention on PAP adherence and PROs. Additionally, incorporating objective measures of sleep quality, such as polysomnography, could provide a more comprehensive assessment of the intervention's effects. Further research should also investigate the specific components of the SAM clinic model that contribute most to its success, enabling optimization and broader implementation of this group-based approach in clinical practice. In particular, exploring strategies for sustained patient engagement and addressing logistical barriers (e.g., scheduling, staffing needs, and resource allocation) will be crucial to ensuring the scalability and long-term viability of the SAM intervention. Cost-effectiveness analyses and adaptations for virtual or hybrid formats may also promote wider adoption. Evaluating the intervention’s feasibility and cost-effectiveness is equally important, particularly given the multidisciplinary staffing and resources required for SAM sessions. Formal economic analyses and pilot implementations in diverse healthcare settings could help determine whether improved PAP adherence and patient outcomes offset these upfront costs, ultimately guiding wider adoption.

While the SAM clinic group demonstrated a numerically higher ESS score at one month, no significant differences were observed between groups for this or other patient-reported outcomes. The observed trends suggest a potential benefit of the SAM intervention, warranting further research in a larger, adequately powered study. The multidisciplinary, group-based approach of the SAM clinic provided comprehensive support and education, which may have contributed to the observed trends. These findings suggest that the SAM clinic process could be beneficial in supporting patients with PAP therapy, though further research with larger sample sizes is needed to confirm its effectiveness. The SAM clinic model emphasizes the importance of providing structured, multidisciplinary support to enhance patient engagement and adherence to PAP therapy.

## Supplementary Information

Below is the link to the electronic supplementary material.

Supplementary file1 (DOCX 227 KB)

## Data Availability

The datasets generated and analyzed during the current study are available from the corresponding author on reasonable request.

## Abbreviations

OSA: Obstructive Sleep Apnea
PAP: Positive Airway Pressure
CMS: Centers for Medicare & Medicaid Services
ESS: Epworth Sleepiness Scale
PHQ-9: Patient Health Questionnaire-9
PROMIS: Patient-Reported Outcomes Measurement Information System
AHI: Apnea–Hypopnea Index
REI: Respiratory Event Index
RDI: Respiratory Disturbance Index
SAM: Sleep Apnea Management
RCT: Randomized Controlled Trial
ICU: Intensive Care Unit
DME: Durable Medical Equipment
HbA1c: Hemoglobin A1c
COVID-19: Coronavirus Disease 2019
