# Supplementary material for: Impact of a multidisciplinary sleep apnea management group clinic on positive airway pressure adherence and patient-reported outcomes: a randomized controlled trial
Source: Sleep Breath. 2025 Apr 4;29(2):149. doi: 10.1007/s11325-025-03319-x (PMC11971166; doi:10.1007/s11325-025-03319-x)
Supplement: Supplementary file 1 — Supplementary file1 (DOCX 227 KB) [file 11325_2025_3319_MOESM1_ESM.docx]

Supplements

**Supplementary** **Table 1.** Baseline Survey Scores by Group

| **Table 2.5. Baseline Summary - Participant Survey by group** | | | | | | | |
| --- | --- | --- | --- | --- | --- | --- | --- |
|  | **Overall (N=56)** | | **Randomized to SAM Clinic (N=26)** | | **Randomized to Usual Care (N=30)** | |  |
| **Factor** | **N** | **Statistics** | **N** | **Statistics** | **N** | **Statistics** | **Standardized Difference** |
| ESS Total Score v2 | 56 | 10.0[6.5,14.0] | 26 | 8.5[5.0,12.0] | 30 | 11.5[8.0,16.0] | 0.57 |
| PHQ-9 Total Score v2 | 56 | 10.0[6.0,13.0] | 26 | 9.5[6.0,13.0] | 30 | 10.0[6.0,13.0] | 0.073 |
| PROMIS Physical T-Score | 56 | 42.3[37.4,47.7] | 26 | 41.1[37.4,50.8] | 30 | 42.3[37.4,44.9] | 0.18 |
| PROMIS Mental T-Score | 56 | 43.5[36.3,48.3] | 26 | 43.5[36.3,48.3] | 30 | 43.5[36.3,48.3] | 0.061 |
| PROMIS Anxiety T-Score | 55 | 55.8[40.3,61.4] | 26 | 51.2[40.3,61.4] | 29 | 55.8[48.0,61.4] | 0.11 |
| PROMIS Fatigue T-Score | 56 | 58.3[52.0,64.7] | 26 | 59.3[52.0,66.9] | 30 | 58.3[52.0,64.7] | 0.11 |
| PROMIS SRI T-Score | 56 | 53.4[48.5,62.0] | 26 | 53.4[47.9,62.0] | 30 | 52.9[49.1,64.0] | 0.092 |
| Statistics presented as N (column %).^[[1]](#footnote-1)^ | | | | | | | |

**Supplementary Figure 1.** Forest Plot of Between-Group Differences at 1 and 3 Months


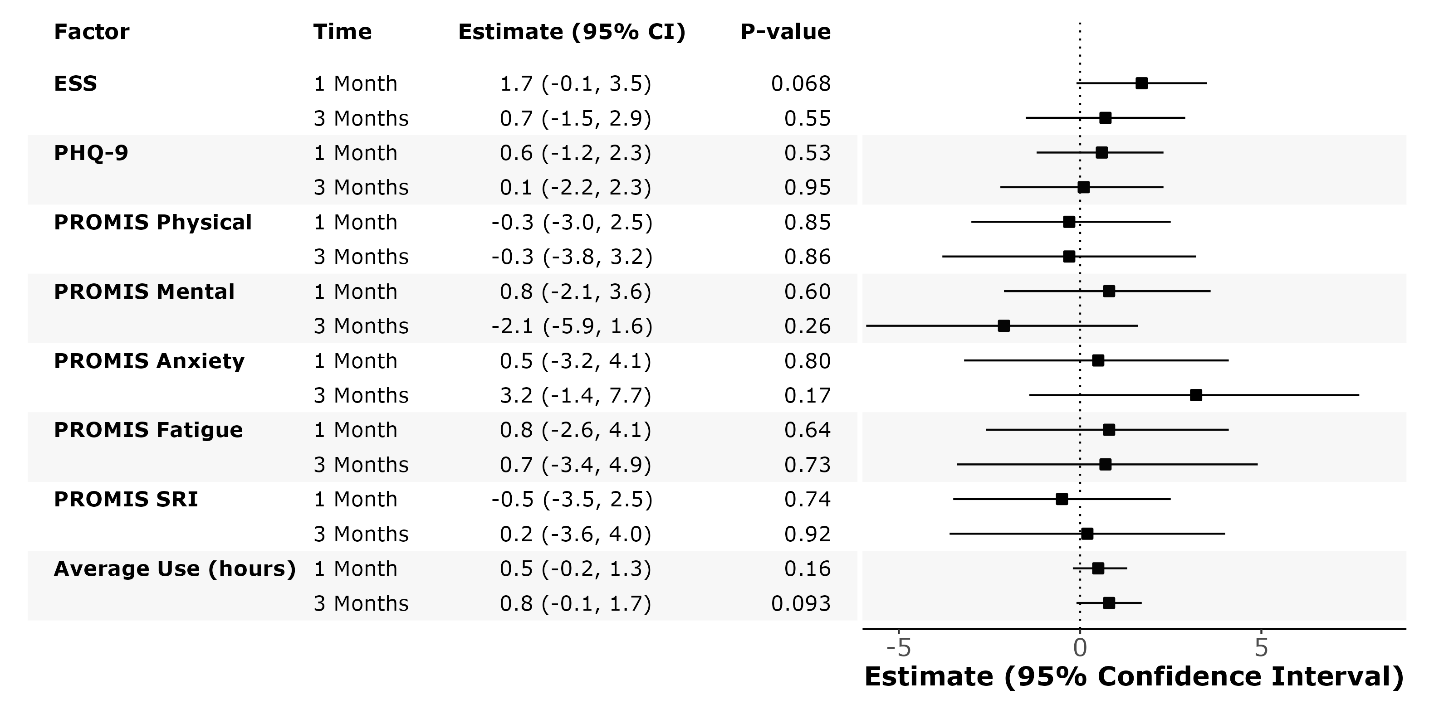
^[[2]](#footnote-2)^

1. Values are presented as median [IQR] or mean (SD) unless otherwise indicated. N = number of participants with valid data for each measure. The “Standardized Difference” quantifies the difference between the SAM clinic and usual care groups relative to the pooled standard deviation. ESS = Epworth Sleepiness Scale; PHQ-9 = Patient Health Questionnaire-9; PROMIS = Patient-Reported Outcomes Measurement Information System (Physical, Mental, Anxiety, Fatigue, and Sleep-Related Impairment [SRI] T-scores). [↑](#footnote-ref-1)
2. This figure shows the estimated between-group differences (SAM clinic minus usual care) at each time point, with 95% confidence intervals (CIs). Factors include the Epworth Sleepiness Scale (ESS), Patient Health Questionnaire-9 (PHQ-9), PROMIS domains, and average PAP usage hours. Horizontal bars represent 95% CIs, and the vertical dashed line marks zero difference. Further numerical detail can be found in Table 4 of the main text. [↑](#footnote-ref-2)
